# Work and the public understanding of science

**DOI:** 10.1177/09636625231203478

**Published:** 2023-10-21

**Authors:** Robert M. Kunovich

**Affiliations:** The University of Texas at Arlington, USA

**Keywords:** interest, science knowledge, science work, trust, work complexity

## Abstract

This study examines whether engaging in science work and work that is substantively complex (e.g. requiring independent thought and judgment) is related to interest in science, science knowledge, and confidence in the scientific community in the United States. It also examines whether the conditions of work mediate the relationship between education and these science-related outcomes. Occupation-level data from O*NET are merged with survey data from the General Social Survey. Results indicate that science work is related to interest in science and science knowledge and that work complexity is related to confidence in the scientific community. Results offer only limited evidence of mediation—science work mediates the relationship between educational attainment and science knowledge but not the relationships involving interest or confidence. In sum, results indicate that the conditions of work are associated with science attitudes, and that researchers should examine these connections in future research.

## 1. Introduction

The public’s understanding of science has been an area of concern and focused research since the 1960s (Bauer et al., 2007). Researchers and policymakers have expressed concern over low levels of knowledge as well as negative orientations, such as a lack of support for the public funding of research (Motta, 2019), low interest (Jensen and Buckley, 2014), and distrust of science (Gauchat, 2012). Attention has expanded to include a focus on the scientific community’s orientations toward the public and efforts to build trust on both sides (Bauer et al., 2007). Research on these topics has been informed by several paradigms, which have focused on public deficits (e.g. how low levels of science knowledge might influence anti-science attitudes in the general public), the public’s contextualized understanding of science (i.e. how knowledge about politics and power influences science attitudes), and expert deficits (i.e. how distrust of the public among experts contributes to the lack of public trust in science; see Bauer et al., 2007 and Sturgis and Allum, 2004 for reviews).

This article focuses on the public understanding of science (PUS)—that is, interest in science, science knowledge, and confidence in the scientific community. PUS research has, as of late, focused relatively more attention on issues related to trust. A main goal has been to better understand how trust in science is related to religiosity (Ecklund and Scheitle, 2017; Evans and Evans, 2008; Noy and O’Brien, 2018; O’Brien and Noy, 2015; Perry et al., 2021; Sherkat, 2017) and political identification (Gauchat, 2012; Motta, 2018; Rekker, 2021). A great deal of research focuses on the relationships between trust and vaccinations (Goldenberg, 2021; Yaqub et al., 2014) and climate science (Fage-Butler et al., 2022).

When researchers and policymakers search for solutions to low knowledge and a lack of interest and trust, they often point to the need to better educate the public, through schooling, but also through more effective communication with the public through media (Lee and Lee, 2022; Noy and O’Brien, 2019; Slovic, 2000). It is hoped that schooling helps people to learn basic scientific facts and procedures but also helps them to develop the critical thinking skills needed to better understand science throughout their life. Understanding science, after all, requires cognitive skills (Rutjens et al., 2018). The relationship between media and PUS is complex because media determines what information is shared and how it is shared and because the reception of information varies due to interest, ability, and identity (see Arnoldi, 2009).

A missing piece of the PUS puzzle, however, may be the role of work. We would certainly expect those working in science-related occupations to have different attitudes toward science. They are, for example, exposed to new discoveries that may increase the desire to learn more. Work in other occupations, though, may also be related to science attitudes. Even in non-STEM fields, people engage in complex work with information and people. Some, for example, make important decisions after gathering and interpreting information from different sources. Others settle disputes between co-workers or among the public. Perhaps, the practice of complex work provides an opportunity after the completion of formal education to further develop the cognitive skills needed to understand science. It may also encourage open-mindedness that shapes how people view the messy and nonlinear process of generating scientific knowledge.

In this article, I examine conditions of work—this is, the substantive complexity of work and the use of scientific rules and methods to solve problems at work—and their relationship with PUS. I examine whether the conditions of work are associated with interest, knowledge, and confidence. I also examine whether the conditions of work mediate the relationship between educational attainment and PUS. If the conditions of work are relevant, it may help us to better understand how science attitudes are shaped throughout the life course and to identify receptive partners for increased public engagement.

## 2. Theoretical background and literature review

### Cognitive ability, education, and media

While much research focuses on political and religious identity as primary sources of attitudes toward science, some have also argued that cognitive ability impacts the public understanding of science. Rutjens et al. (2018), for example, argue that many scientific theories defy common sense understandings and that training may be required to understand scientific theory and methodology (see also McCauley, 2011; Shtulman, 2017). In the absence of training, some people may instinctively rely on heuristics, such as essentialism or teleological reasoning (Rutjens et al., 2018). Heuristics are shortcuts that our brains use automatically to simplify complex situations and to make decisions (Kahneman, 2011). Relying on these shortcuts, however, opens us up to various biases that can lead to errors (see Tversky and Kahneman, 1974) and creates barriers to understanding science. Essentialist reasoning, for example, suggests that racial identities are rooted in stable genetic and cultural attributes. This type of thinking reifies racial categories, promotes stereotypes, and obscures that racial identification is an ongoing social process linked to power (Dar-Nimrod and Heine, 2011). Others discuss how essentialism, teleological reasoning, and other biases impact the public’s understanding of evolution (Blancke et al., 2012; Evans, 2001) and genetics (Dar-Nimrod and Heine, 2011).

Recent research suggests that cognitive ability influences not only people’s capacity to understand science but other science-related attitudes as well. Diekmann et al. (2017), for example, demonstrate that cognitive ability is related to public perceptions of disagreements between scientists. While scientists view disagreements as fundamental to the scientific process, some members of the public view such disagreements as troubling (Douglas and Wildavsky, 1982; Giddens, 1990). Those with lower cognitive abilities attribute disagreements to incompetence among scientists, while those with higher cognitive abilities attribute them to the complexities and randomness of the topic of study (Dieckmann et al., 2017). Similarly, Motta (2018) shows that verbal intelligence is associated with confidence in the scientific community and trust in experts and intellectuals (i.e. intellectualism versus anti-intellectualism).

Many have argued that better science education can improve the situation. Improvements could come through new initiatives in schooling but also through public education campaigns in traditional and social media. Research does suggest that education is related to the public understanding of science. Bak (2001), for example, shows that education and science knowledge have independent relationships with science attitudes and that science attitudes do not differ across educational content (e.g. science and engineering majors compared to humanities, social science, and other majors). Achterberg et al. (2017) demonstrate that educational attainment is positively associated with trust in scientific institutions but not trust in scientific methods and that the gap between trust in institutions and methods is larger for those with less education. Our ability to shape public attitudes toward science through media, however, is limited because media organizations amplify or de-amplify certain topics and promote specific meanings, and there is great diversity in media sources, and information from media is interpreted in different ways depending on people’s backgrounds and ingrained beliefs (see Arnoldi, 2009; De Silva-Schmidt et al., 2022; Grien and MacNeil, 2022; Rekker, 2021).

### The conditions of work: Science work and complex work

While new initiatives through schooling and science education through media may shape PUS, research has largely overlooked the possible role of work. The work that we do provides additional opportunities to develop and practice skills that might influence our understanding of science and the scientific process. This may be true for those working in science-related fields but also for those working in non-STEM fields requiring complex work (e.g. critical thinking, collaboration, conflict resolution, independence).

Data from the US Bureau of Labor Statistics indicates that about 12% of workers are employed in a STEM field (Landivar, 2013; US Bureau of Labor Statistics, 2022). While people with greater interest in and knowledge of science are likely drawn to science-related occupations in the first place, working in a STEM field may extend interest, knowledge, and confidence for a variety of reasons. First, people working in science-related fields are at the forefront of scientific research and development, which can expose them to new ideas, discoveries, and technologies and create a desire to learn more. Second, scientific research is collaborative and often interdisciplinary. Collaboration may expose people to new ideas as well as to other people who are interested in and passionate about science. Interdisciplinary collaboration may increase knowledge because it promotes cross-disciplinary learning. Third, those engaged in science work are often trying to develop practical solutions to solve problems. Doing work that is believed to benefit society may increase people’s interest and commitment to this work. Finally, working in a science-related field requires extensive education and training, which may provide a foundation of knowledge and an appreciation for the importance of science. While most of these explanations suggest that there is a direct relationship between the conditions of work and science attitudes, this last possibility suggests that working in a science-related field may mediate the relationship between education and PUS—that is, the conditions of work may transmit some of the relationships between education and interest, knowledge, and confidence.

Those working in STEM fields, however, are not the only ones who develop and practice cognitive skills that might shape science attitudes. Many other people are employed in non-STEM fields that require complex work. Some work is complex with respect to information, reasoning, and decision-making because it requires people to collect and analyze information to choose the best solution to complicated problems. Emergency management directors, for example, are expected to develop plans for and to train others to respond to various natural and human-made disasters, such as hurricanes and hostage situations. Perhaps, such work helps people to further develop critical thinking skills and to avoid reliance on heuristics that impede their ability to understand science. Even though they may not be using science to generate new knowledge themselves, such work may also require collaboration with scientists as well as the possession of a baseline of scientific knowledge. Other work is complex with respect to people because it requires handling complaints, settling disputes, and negotiating with others. Human resources managers, for example, are required to take the time to understand others’ points of view and to find workable solutions to conflicts. Such work may play a role in shaping how people view disagreements between scientists; for example, maybe they are more likely to view disagreements as normal. Finally, higher levels of education are often required to work in complex occupations. Those engaged in complex work may have more interest, knowledge, and confidence in science due to this background. This last possibility suggests that working in a complex occupation may mediate the relationship between education and PUS. In sum, engaging in complex work—such as gathering and processing complex information, problem-solving, creative thinking, and interacting with others—may play some role in shaping people’s orientations toward science even for those not working in a STEM field.

Although the conditions of work have largely been overlooked in studies related to PUS, research demonstrates that work influences personality in important ways. Conditions of work, such as the complexity of work and freedom from supervision, impact various dimensions of psychological functioning, such as intellectual flexibility (Kohn and Schooler, 1978; Kohn and Slomczynski, 1993; Kohn et al., 1990). Kohn et al. (1990), for example, show that those who engage in complex work are better able to adapt their thinking when confronted with new situations, ideas, and problems. Other observed consequences from complex work include being trustful of others, having a sense of control and believing that your actions can make a difference, making decisions even if they go against the majority, and making ethical choices even when others are not watching (Kohn et al., 1990). These characteristics—flexibility, adaptiveness, and openness to change—would seem to work against the reliance on mental shortcuts that impede an understanding of science (e.g. McCauley, 2011; Rutjens et al., 2018; Shtulman, 2017). They may also impact the way people view the process of knowledge creation in the sciences, which can sometimes appear chaotic and nonlinear.

In sum, existing research suggests that the primary drivers of the public understanding of science are political and religious identities and cognitive ability. While there is research examining the effects of education on PUS or the efficacy of various media communication strategies, the conditions of work as a possible source of interest, knowledge, and confidence have largely been overlooked. Research on work and personality, however, suggests that those working in occupations requiring complexity may also develop the cognitive skills needed to understand science and there are a variety of reasons to expect those working in STEM fields to have greater interest, knowledge, and confidence in science. In this study, I examine whether the conditions of work are associated with PUS and whether the conditions of work mediate the relationship between education and PUS.

## 3. Hypotheses

H1. The conditions of work have a direct relationship with PUS:
 A. Those who work in occupations requiring complexity and the use of science express greater interest in science.
 B. Those who work in occupations requiring complexity and the use of science possess greater science knowledge.
 C. Those who work in occupations requiring complexity and the use of science are more likely to express confidence in the scientific community.
H2. The conditions of work mediate the relationships between educational attainment and PUS:
 A. Educational attainment has an indirect relationship with interest through the conditions of work.
 B. Educational attainment has an indirect relationship with knowledge through the conditions of work.
 C. Educational attainment has an indirect relationship with confidence through the conditions of work.

## 4. Methods

### Data

Survey data are from the 2018 General Social Survey (GSS) cross-section (Smith et al., 2019), which is a nationally representative sample of adults in the United States. The 2018 GSS cross-section is one part of a larger series dating back to 1972. Each GSS wave contains a repeating set of core items as well as additional items on special topics, such as science knowledge and attitudes. Data collection is performed by the National Opinion Research Center at the University of Chicago and is funded by the National Science Foundation. Data from the GSS data series are well documented online at https://gss.norc.org/.

Data for the 2018 GSS Cross-section were collected between April 12 and November 11, 2018, using computer-assisted personal interviewing techniques (CAPI). The total sample size for the 2018 GSS cross-section is 2348. Given the focus on conditions of work, analyses are limited to those respondents who are working or who have ever worked (2258 of the original 2348 respondents). While newer GSS Cross-sections (2021 and 2022) and panel data (2016–2020) are now available, these samples are missing important science-related variables needed for this analysis. The cross-sectional data from 2018, which are used in this analysis, are also limited in one important way. The survey was split into three ballot groups A, B, and C (the sample sizes for the three ballots in 2018 are A-785, B-774, and C-789). Some important variables were measured in only two of three ballots, reducing the number of cases for some analyses and limiting the use of some variables as controls. I provide more details about this below in the measurement section as well as in the final paragraph of the results. I discuss the limitations of the cross-sectional data in the section “Discussion and conclusion”.

Occupation-level data measuring work complexity and the use of science in the occupation are from version 24.2 of the O*NET Resource Center database (National Center for O*NET Development, 2022). Researchers at the Center for O*NET development, which is part of the US Department of Labor, conduct interviews with job incumbents and experts covering many concepts related to worker characteristics, worker requirements, experience requirements, occupational requirements, workforce characteristics, and occupation-specific information (these are the six dimensions of the O*NET Content Model).

The O*NET data used to measure work complexity and science work (described below) are available at the aggregate level for detailed occupations—that is, publicly available O*NET data files (online at the O*NET Resource Center) list mean values and other basic statistics for each item at the level of detailed occupations (e.g. the mean level of freedom from supervision for all job incumbents working as financial managers). While both O*NET and GSS data files contain identification variables that indicate occupation (e.g. “financial manager”), they rely on different coding schemes. The detailed occupation codes in the O*NET data are referred to as O*NET SOC codes while the GSS utilizes occupation codes from the 2010 Census (e.g. the numeric codes for “financial manager” are 11-3031 in the O*NET SOC scheme and 0120 in the 2010 Census scheme used in the GSS). To link the occupation data from O*NET to specific people in the GSS, it is necessary to convert one occupational coding scheme into another (i.e. O*NET SOC to 2010 Census). This is possible using crosswalks (the Census Bureau publishes such crosswalks on its web page). Cross-walks link occupational titles (e.g. financial manager) and occupational codes (e.g. 11-3031 and 0120) across different coding schemes (e.g. O*NET SOC and 2010 census). These cross-walks allow researchers to recode occupation codes from one scheme to another. After doing so, it is possible to merge the files such that the occupation-level data from O*NET are assigned to each case in the GSS.

### Measurement

I use data from the GSS to measure three dependent variables including interest in science, science knowledge, and confidence in the scientific community as well as education and the control variables. I measure *interest in science* with a standardized scale composed of four variables (alpha = .78). The four variables, each with three ordinal response choices (not at all interested, moderately interested, or very interested), indicate interest in issues about (1) new scientific discoveries, (2) new inventions and technologies, (3) new medical discoveries, and (4) space exploration. I measure *science knowledge* with the percentage correct out of 11 true or false questions. This measure is often referred to as the “Oxford Scale” (see Allum et al., 2008). The 11 true or false questions cover basic science facts and methodology. I code answers of “don’t know” as incorrect. I omit two other possible items focusing on evolution and the Big Bang because they are just as likely to measure religious orthodoxy as science knowledge (Evans, 2011). The third dependent variable, *confidence in the scientific community*, is measured with a single question: “I am going to name some institutions in this country. As far as the people running these institutions are concerned, would you say you have a great deal of confidence, only some confidence, or hardly any confidence at all in them? Scientific Community.” I recoded this into a dichotomous variable with categories of (1) “hardly any/only some” and (2) “a great deal” because only 6.6% of respondents selected “hardly any.” It should be noted that trust is multidimensional—it includes trust in institutions as well as trust in the methods of science (Achterberg et al., 2017). Due to data availability, my focus is on trust in the people running scientific institutions. Measures for interest in science and science knowledge are available for respondents assigned to ballots A and B (with a maximum possible sample size of 1559), while measures for confidence are available for respondents assigned to ballots B and C (with a maximum possible sample size of 1563).

Two measures of *education* are available in the GSS data: educational attainment (i.e. the highest degree earned) and the number of years of schooling completed. The categories for educational attainment include: less than high school, high school, associate’s degree/junior college, bachelor’s degree, and graduate degree. I rely on educational attainment because of its direct impact on occupational outcomes. A basic requirement for some jobs, for example, is to have a bachelor’s degree. In addition, there is variability in the years of schooling for people with the same degree (e.g. it takes some people longer to complete a bachelor’s degree). I do, however, present models that include years of schooling to evaluate the robustness of the results.

With respect to *control variables*, previous research reviewed above suggests that religious identity (e.g. O’Brien and Noy, 2015), political identity (e.g. Rekker, 2021), and verbal intelligence (e.g. Motta, 2018) are important predictors of science attitudes. I control for religious identity using the RELTRAD coding strategy which differentiates between evangelical Christians, mainline Protestants, Black Protestants, Catholics, Jews, those of some other faith, and those with no religious affiliation (see Stetzer and Burge, 2016). I measure political identity with a set of political party dummy variables: Democrat (the reference group), Independent, Republican, and other party. The score on a 10-question vocabulary test measures verbal intelligence (grand-mean centered).

Previous research also indicates that there are important differences in science attitudes across sex, age, and race and ethnicity (e.g. Gauchat, 2012). I therefore include as controls a sex dummy variable, age in years (grand-mean centered), and race and ethnicity (with dummy variables for Black, Hispanic, and other and White as the omitted reference category). Finally, I include a dummy variable indicating whether the respondent works full-time. To be included in the sample, people must either have worked in the past or be currently working (anyone never having worked lacks an occupation code and, therefore, cannot have scores on work complexity and science work). The dummy variable differentiates between those currently working full-time and everyone else (e.g. part-time workers, those who are retired).

Using occupation-level data from O*NET I include two indicators of work conditions. The first is *work complexity*, which is measured with a standardized variable. This measure covers multiple dimensions of work complexity including the complexity of information (e.g. the level and importance of observing, receiving, and otherwise obtaining information from all relevant sources), the complexity of mental activities (e.g. analyzing information and evaluating results to choose the best solution and solve problems), the complexity of interacting with others (e.g. encouraging and building mutual trust, respect, and cooperation among team members), and the independence of work (i.e. freedom from supervision). The second occupation-level variable is *science work*—that is, the extent to which scientific rules and methods are used to solve problems while working in the occupation. The science work variable is also measured in z scores. It is based on a single item in the O*NET data. The bivariate correlation between the complexity of work and science work is .55. A detailed discussion of the measurement of work complexity and science work is available in the supplemental materials.

Except for verbal intelligence, all independent variables are available for respondents completing ballots A, B, and C. Verbal intelligence is only available for those completing ballots A and B (with a maximum possible sample size of 1559). Basic descriptive statistics as well as information on the extent of missing data are available in the supplemental materials.

### Analytic technique

In the analyses below, I regress the conditions of work on educational attainment and the control variables (without a relationship, the conditions of work could not mediate the relationship between education and PUS). After that, I estimate a set of linear models for interest and knowledge and a set of binary logistic models for confidence. I present three models for each science-related outcome. The first shows the slopes for educational attainment and control variables. In the second model, I step into work complexity and science work to identify differences in the educational attainment slopes (to assess possible mediation). In the third model, I replace educational attainment with years of schooling to assess the robustness of the results. Finally, I run additional path models to estimate the indirect effects of educational attainment through the conditions of work. Given that the survey respondents are nested within occupations (all people working/having worked in the same occupation are assigned the same score on work complexity and use of science), I present robust (i.e. cluster-corrected) standard errors for all models. All models were estimated in STATA.

## 5. Results

For the conditions of work to mediate the relationship between education and PUS, there must be a relationship between education and the conditions of work. I present results from two linear regressions in Table 1 to determine whether such relationships exist. The dependent variables are the complexity of work and science work (both are measured in z scores). Independent variables include educational attainment (i.e. highest degree earned) and the set of control variables used in all analyses. Results indicate that there is a relationship between educational attainment and the conditions of work. Those with higher degrees tend to work in occupations that are more complex and that require the use of scientific rules and methods to solve problems. Those with bachelor’s degrees, for example, score about 1 and .5 z scores higher on the complexity of work and science work, respectively (slopes = .999 and .531).

**Table 1.** Complexity of work and science work with the highest degree: linear regressions.

	Complexity of work (z)		Science work (z)	
	Slope	Robust SE	Slope	Robust SE
Highest degree (ref = less than HS)				
High school	.271*	.089	.045	.055
Junior college	.756*	.160	.356*	.192
Bachelor’s degree	.999*	.110	.531*	.106
Graduate degree	1.333*	.130	.915*	.167
*Control variables*				
Sex (male = 1)	.093	.084	.134	.096
Age (Centered)	.006**	.002	.003	.001
Race and ethnicity (ref = White)				
Black	−.083	.089	−.098	.089
Hispanic	−.178**	.067	−.150**	.069
Other	.081	.101	.048	.099
Rel. identity (ref = Evangelical)				
Mainline	.025	.075	−.012	.087
Black Protestant	.034	.109	−.093	.115
Catholic	.032	.061	−.023	.060
Jewish	.241	.144	.001	.231
Other faith	.047	.122	−.031	.098
No affiliation	−.038	.058	.002	.067
Political party (ref = Democrat)				
Independent	.082	.067	.011	.064
Republican	.185**	.087	.060	.098
Other party	.272	.152	.095	.157
Working full-time (yes = 1)	.220**	.054	.108**	.050
Vocabulary test score (centered)	.052**	.013	.021	.014
Intercept	−.758	.135	−.359	.093
N	1415		1415	
F	15.820		4.750	
DF 1	20		20	
DF 2	310		310	
Prob > F	.000		.000	
R-squared	.298		.150	
Root MSE	.837		.866	

Note. HS: high school; DF: degrees of freedom; MSE: mean squared error; SE: standard error.

* *p* < .05 (one-tailed); **p < .05 (two-tailed).

I present results for interest in science in Table 2 (interest is measured in z scores). I show the relationship between educational attainment and interest in science net of the control variables in Model 1. I step into the work variables in Model 2 to look for evidence of mediation. I replaced educational attainment with years of schooling in Model 3 to evaluate the robustness of the results. The results indicate that science work is associated with interest in science—those who work in occupations requiring the use of scientific rules and methods to solve problems tend to report greater interest in science net of educational attainment and the control variables (slope = .083). This relationship exists regardless of which education variable is included in the model. The complexity of work, however, is not related to interest in science. While significant, the strength of the relationship between science work and interest is moderate at best. The predicted difference in interest for those with the highest and lowest possible scores on science work is about .5 *z* scores (the range for science work is 6.33; 6.33 × .083 = .525).

**Table 2.** Interest in Science (in z Scores): Linear Regressions.

	Model 1		Model 2		Model 3	
	Slope	Robust SE	Slope	Robust SE	Slope	Robust SE
Highest degree (ref = less than HS)						
High school	.337*	.101	.335*	.104		
Junior college	.446*	.126	.416*	.131		
Bachelor’s degree	.509*	.113	.455*	.121		
Graduate degree	.660*	.127	.599*	.139		
Years of education (centered)					.049*	.013
Complexity of work (z score)			.003	.038	.002	.038
Science work (z score)			.083*	.031	.080*	.031
*Control variables*						
Sex (male = 1)	.199**	.058	.168**	.057	.164**	.057
Age (Centered)	.002	.002	.002	.002	.002	.002
Race and ethnicity (ref = White)						
Black	.058	.115	.044	.119	.025	.120
Hispanic	.142	.085	.139	.087	.129	.084
Other	.040	.111	.054	.111	.042	.113
Rel. identity (ref = Evangelical)						
Mainline	.068	.096	.073	.096	.081	.097
Black Protestant	−.024	.165	.030	.169	.024	.170
Catholic	.022	.094	.029	.095	.022	.096
Jewish	.023	.209	−.017	.212	−.040	.205
Other faith	.229	.141	.236	.141	.249	.142
No affiliation	.317**	.091	.312**	.094	.318**	.094
Political party (ref = Democrat)						
Independent	−.024	.070	−.001	.072	.013	.073
Republican	−.093	.084	−.055	.085	−.053	.085
Other party	.136	.186	.131	.194	.114	.193
Working full-time (yes = 1)	−.108	.064	−.122	.066	−.114	.067
Vocabulary test score (centered)	.068**	.016	.070**	.016	.067**	.016
Intercept	−.493	.145	−.473	.150	−.120	.106
N	1098		1063		1062	
F	7.250		6.890		7.620	
DF 1	20		22		19	
DF 2	295		277		277	
Prob > F	.000		.000		.000	
R-squared	.095		.099		.098	
Root MSE	.954		.952		.949	

Note. HS: high school; DF: degrees of freedom; MSE: mean squared error; SE: standard error.

* *p* < .05 (one-tailed); **p < .05 (two-tailed).

Results also indicate that educational attainment is associated with interest in science. Those with a bachelor’s degree, for example, score about .5 *z* scores higher on interest compared to those with less than a high school degree (slope = .455). There does not, however, appear to be any evidence that the conditions of work mediate the relationship between educational attainment and interest in science. After controlling for the conditions of work, there is little difference in the educational attainment slopes (i.e. between Models 1 and 2). This conclusion is confirmed in the results from a separate structural equation model for which direct, indirect, and total effects are available (I present the direct, indirect, and total effects for educational attainment in Table 3). None of the indirect effects are statistically significant. In sum, there is partial support for hypothesis 1A because only science work is associated with interest. There is no support for hypothesis 2A.

**Table 3.** Educational attainment: direct, indirect, and total effects.^a^

	Direct		Indirect		Total	
	Slope	Robust SE	Slope	Robust SE	Slope	Robust SE
DV = Interest in science						
Highest degree (ref = less than HS)						
High school	.335*	.116	.001	.010	.336*	.116
Junior college	.416*	.145	.031	.028	.447*	.142
Bachelor’s degree	.455*	.130	.050	.035	.505*	.124
Graduate degree	.599*	.148	.085	.049	.684*	.140
DV = Science knowledge						
Highest degree (ref = less than HS)						
High school	7.613*	1.920	.226	.216	7.839*	1.932
Junior college	7.342*	2.513	1.112*	.501	8.454*	2.471
Bachelor’s degree	14.480*	2.347	1.658*	.642	16.138*	2.270
Graduate degree	15.165*	2.754	2.537*	.876	17.702*	2.642

Note. DV: dependent variable; HS: high school; SE: standard error.

a Direct, indirect, and total effects were estimated in STATA using the SEM procedure with robust standard errors. Results are shown only for educational attainment, but the models contain the same variables as reported in Tables 1, 2, and 3. It is not possible to estimate indirect effects in STATA using the generalized structural equation modeling (GSEM) procedure that is needed for the binary outcome, and confidence in the scientific community.

* *p* < .05 (one-tailed).

I present results for science knowledge in Table 4. The results indicate that science work is associated with science knowledge—those who work in occupations requiring the use of scientific rules and methods to solve problems tend to have greater science knowledge, net of educational attainment, and the control variables. For example, those differing by 1 *z* score on science work are expected to differ by about 1.3% on the science knowledge test (slope = 1.262). This relationship exists regardless of which education variable is included in the model. While significant, the strength of the relationship between science work and knowledge is also modest. The predicted difference in knowledge for those with the highest and lowest possible scores on science work is about 8 percentage points (the range for science work is 6.33; 6.33 × 1.232 = 7.988).

**Table 4.** Science knowledge (in percentages): linear regressions.

	Model 1		Model 2		Model 3	
	Slope	Robust SE	Slope	Robust SE	Slope	Robust SE
Highest degree (ref = less than HS)						
High school	8.316*	1.730	7.613*	1.673		
Junior college	9.306*	2.242	7.342*	2.246		
Bachelor’s degree	16.601*	2.141	14.480*	2.150		
Graduate degree	18.675*	2.563	15.165*	2.610		
Years of education (centered)					1.783*	0.225
Complexity of work (z score)			.956	.670	.755	.677
Science work (z score)			1.262*	0.599	1.200*	.622
*Control variables*						
Sex (male = 1)	3.657**	1.076	3.244**	1.091	3.311**	1.092
Age (Centered)	−.199**	.033	−.202**	.033	−.182**	.032
Race and ethnicity (ref = White)						
Black	−11.162**	2.053	−11.425**	2.107	−11.808**	2.047
Hispanic	−4.732**	1.618	−4.233**	1.631	−4.208**	1.588
Other	−4.445	2.605	−4.897	2.689	−4.771	2.606
Rel. identity (ref = Evangelical)						
Mainline	3.085	1.620	3.561**	1.632	3.615**	1.631
Black Protestant	1.726	2.647	2.403	2.683	2.317	2.585
Catholic	−.127	1.391	−.004	1.396	.092	1.373
Jewish	−3.689	3.379	−4.129	3.438	−5.281	3.479
Other faith	4.464**	2.182	4.758**	2.174	5.081**	2.121
No affiliation	1.852	1.534	2.127	1.576	2.035	1.601
Political party (ref = Democrat)						
Independent	−.778	1.289	−1.016	1.307	−.242	1.327
Republican	2.051	1.380	1.521	1.414	1.681	1.426
Other party	1.777	2.247	.875	2.226	.325	2.140
Working full-time (yes = 1)	−.140	1.105	−.093	1.127	.232	1.113
Vocabulary test score (centered)	3.594**	.296	3.558**	.303	3.415**	.302
Interest in science (*z* score)	2.513**	.585	2.475**	.592	2.315**	.601
Intercept	57.267	2.233	58.790	2.179	67.212	1.550
N	1082		1048		1047	
F	49.100		40.810		43.980	
DF1	21		23		20	
DF2	294		277		277	
Prob > F	.000		.000		.000	
R-squared	.401		.404		.412	
Root MSE	16.496		16.441		16.266	

Note. HS: high school; DF: degrees of freedom; MSE: mean squared error; SE: standard error.

* *p* < 0.05 (one-tailed); **p < 0.05 (two-tailed).

Results also demonstrate that educational attainment is associated with science knowledge. Those with a bachelor’s degree, for example, score about 14 percentage points higher on the science knowledge test (slope = 14.480). There is also evidence that science work partially mediates the relationship between educational attainment and science knowledge. While educational attainment remains significant in model 2 after controlling for the conditions of work, the gaps between educational attainment groups are somewhat diminished after controlling for the conditions of work (e.g. the slope for a bachelor’s degree decreases from 16.601 to 14.480 from model 1 to model 2). Estimates from a separate structural equation model (presented in Table 3) indicate that these indirect effects are significant. While educational attainment has a direct relationship with science knowledge, it also has an indirect relationship with science work. The total effect of having a bachelor’s degree, for example, is 16.138. This means that, on average, those with a bachelor’s degree score about 16 percentage points higher on the science knowledge test as compared to those with less than a high school degree. About 14 percentage points of this gap are a direct result of educational attainment, while 2 percentage points are a result of the relationship between education and science work (education is related to science work, and science work is related to science knowledge). In sum, results provide partial support for hypothesis 1B and partial support for hypothesis 2B.

I present results for confidence in the scientific community in Table 5. Results indicate that the complexity of work is associated with confidence. This is true regardless of which version of education is controlled. Those working in occupations that require greater complexity are more likely to express confidence in the scientific community (odds ratio = 1.139 = e^0.130^). The odds of having a great deal of confidence differ by about 13.9% when comparing people who differ by 1 z score on complexity of work. The predicted probabilities of having a great deal of confidence differ by about 0.13 for those with the lowest and highest scores on work complexity (the predicted probabilities, estimated using the margins command in STATA, range from .37 to .50 across work complexity). Results also indicate that educational attainment is related to confidence in the scientific community. Those with higher degrees are more likely to respond that they have a great deal of confidence in the scientific community. Given the similarity of the logit coefficients in models 1 and 2, there is no evidence that the conditions of work mediate the relationship between educational attainment and confidence. Formal tests are not available in STATA (because the outcome is dichotomous), but mediation seems very unlikely given the similarity in logit coefficients across models 1 and 2. In sum, the results from Table 5 provide partial support for Hypothesis 1C but not 2C.

**Table 5.** Confidence in the scientific community: binary logistic regressions.

	Model 1		Model 2		Model 3	
	Logit	Robust SE	Logit	Robust SE	Logit	Robust SE
Highest degree (ref = less than HS)						
High school	.451*	.215	.437*	.225		
Junior college	.577*	.281	.533*	.287		
Bachelor’s degree	1.405*	.237	1.321*	.254		
Graduate degree	1.598*	.299	1.532*	.330		
Years of education (centered)					.131*	.026
Complexity of work (*z* score)			.130*	.076	.178*	.076
Science work (z score)			.030	.072	.051	.072
*Control variables*						
Sex (male = 1)	.513**	.114	.521**	.116	.513**	.115
Age (Centered)	−.008**	.004	−.010**	.004	−.009**	.004
Race and ethnicity (ref = White)						
Black	−.653**	.187	−.632**	.191	−.644**	.191
Hispanic	−.147	.195	−.041	.197	−.007	.196
Other	−.212	.222	−.267	.229	−.268	.227
Rel. identity (ref = Evangelical)						
Mainline	.343	.186	.295	.190	.317	.190
Black Protestant	.053	.291	.065	.298	.083	.298
Catholic	.481**	.156	.409**	.159	.419**	.156
Jewish	.276	.457	.234	.457	.272	.440
Other faith	.372	.289	.292	.284	.324	.285
No affiliation	.579**	.162	.569**	.166	.571**	.161
Political party (ref = Democrat)						
Independent	−.359**	.141	−.360**	.144	−.339**	.146
Republican	−.536**	.186	−.547**	.192	−.537**	.190
Other party	−.573	.325	−.578	.323	−.661**	.316
Working full-time (yes = 1)	−.231	.120	−.298**	.125	−.255**	.125
Intercept	−.963	.251	−.889	.263	−.245	.164
N	1427		1388		1387	
Wald chi-square	152.560		170.600		125.93	
DF	19		21		18	
Prob > chi-square	.000		.000		.000	
Pseudo r-squared	.083		.089		.077	
Log pseudo-likelihood	−900.951		−870.060		−881.127	

Note. HS: high school; DF: degrees of freedom; SE: standard error.

* *p* < .05 (one-tailed); **p < .05 (two-tailed).

It should be noted that the models in Table 5 do not control for the vocabulary test score, interest in science, or science knowledge. Unfortunately, the variables used in this analysis appear in only two of three ballots of the GSS questionnaire. Interest, knowledge, and the vocabulary test scores are only available for respondents who completed ballots A and B, while confidence is only available for those completing ballots B and C. When the model predicting confidence (shown in Table 5) is limited to respondents from ballot B and interest, knowledge, and the vocabulary test scores are controlled, the sample size drops from 1388 to 334 cases. Complexity of work is no longer significant in this model (results can be seen in the supplemental materials). The results then suggest that the complexity of work is associated with confidence, but these results are not robust among a small sample of respondents once other variables are controlled.

## 6. Discussion and conclusion

The primary conclusion is that the conditions of work are associated with the public understanding of science. Science work is associated with both interest in science and science knowledge, while work complexity is associated with confidence in the scientific community. Despite the relationships between education, the conditions of work, and PUS, there is only limited evidence of mediation. The conditions of work do not appear to mediate the relationships between educational attainment and interest in science nor confidence in the scientific community. By contrast, part of the relationship between educational attainment and science knowledge can be attributed to educational attainment’s relationship with science work. That is, those with higher degrees tend to have higher levels of science knowledge in part because they work in occupations requiring the use of science. Even here, though, the direct relationship for educational attainment is much larger than any indirect relationship through work conditions. What does this mean? The relationships between science attitudes with education and the conditions of work are largely independent. We do not see a relationship between educational attainment with interest or confidence because education leads to complex work or science work. Instead, there is some other mechanism connecting education to science attitudes. Moreover, the relationships between science work with interest and complex work with confidence do not simply reflect the more extensive education and training requirements for working in these types of occupations.

It is also interesting to note that science work is not associated with confidence. This finding should not, however, lead to the conclusion that there is an epistemological crisis among scientists. The measure of confidence focuses only on one dimension—that is, confidence in the scientific community (i.e. the people running the institutions). Unfortunately, it is not possible to measure another important dimension of confidence, confidence in the methodology of science (see Achterberg et al., 2017). It would be interesting to see if a relationship exists between science work and the methodology of science—one would certainly hope that it does.

Several interesting possibilities exist for future PUS research involving work. First, future research could examine the mechanisms linking the conditions of work with PUS. Kohn and others (e.g. Kohn et al., 1990) demonstrate with panel and cross-national data that work complexity impacts various dimensions of psychological functioning including intellectual flexibility, self-directedness, valuing self-directedness in children, and well-being. Other research in PUS suggests that cognitive ability is an important predictor of science attitudes (Rutjens et al., 2018). These separate contributions to the literature point to factors, such as cognitive ability, intellectual flexibility, and non-authoritarian personality, as likely mechanisms connecting work conditions and PUS. Several other possibilities also exist. Those working in STEM fields often collaborate with other scientists while trying to solve complex problems. Being embedded in a workgroup of scientists and doing work that you believe benefits society may help to explain connections between science work, interest, and knowledge that are independent of prior education and training. Some of those engaged in complex work must settle disputes while considering the validity of competing arguments. Perhaps this type of work influences trust in others more generally or shapes how people view disagreements between scientists.

Second, future research could examine whether the conditions of work interact with other characteristics to shape science attitudes. Existing research, for example, examines whether media generates an education gap in science knowledge. There is some indication that media interacts with education such that the gap between trust in scientific institutions and methods is larger for those with less education (e.g. Achterberg et al., 2017). It would be interesting to see whether the science attitude gaps between religious and political groups differ by the complexity of work or science work. Perhaps, such gaps would be smaller for those engaged in complex work and science work—for example, the confidence gap between Democrats and Republicans might be smaller for Republicans working in science-related fields as compared to Republicans in non-science fields. Additional research is needed to explore the links between education, work complexity, media effects, political and religious identity, and the public understanding of science.

Finally, an important limitation should be addressed—the conclusions are based on an analysis of cross-sectional data. With cross-sectional data, it is impossible to determine whether the conditions of work cause science attitudes, whether science attitudes cause people to work in certain occupations, or if both possibilities are true. It seems plausible that mutual reinforcement exists. For example, people with greater interest in and knowledge of science seek out jobs in STEM fields *and* working in science-related fields further increases interest and knowledge. Unfortunately, it is not possible to use the 2016-2020 GSS Panel to shed light on this issue. Most science-related variables are not available in the second wave of the panel. While it is possible to measure confidence in repeated waves, there is insufficient variation over time for fixed effects panel models to be viable (see Motta, 2019 for an effective use of such a model). It is worth highlighting, however, that this analysis does at least demonstrate an *association* between the conditions of work and PUS. In the future, researchers should collect panel data and attempt to disentangle the direction of these relationships.

In sum, the conditions of work are associated with interest in science, science knowledge, and confidence in the scientific community. These relationships appear to exist independently of educational attainment—that is, the conditions of work do not mediate the relationship between education and PUS. Researchers should include work in future studies of the public understanding of science to build theories that focus on differences and changes in PUS over the life course.

## Supplemental Material

sj-docx-1-pus-10.1177_09636625231203478 – Supplemental material for Work and the public understanding of science Supplemental material, sj-docx-1-pus-10.1177_09636625231203478 for Work and the public understanding of science by Robert M. Kunovich in Public Understanding of Science
