# Supplementary material for: Work and the public understanding of science
Source: Public Underst Sci. 2023 Oct 21;33(3):353–69. doi: 10.1177/09636625231203478 (PMC10958755; doi:10.1177/09636625231203478)
Supplement: sj-docx-1-pus-10.1177_09636625231203478 – Supplemental material for Work and the public understanding of science [file sj-docx-1-pus-10.1177_09636625231203478.docx]

**WORK AND THE PUBLIC UNDERSTANDING OF SCIENCE**

**Robert M. Kunovich**

**Department of Sociology and Anthropology**

**The University of Texas at Arlington**

**Email: Kunovich@uta.edu**

**Supplemental Materials**

**Table of Contents**

- Measurement of the Occupation-level Variables
  - Complexity of Work
  - Science Work
  - Table A.1. Item Details
  - Table A.2. Factor Loadings
  - Table A.3. Bivariate Correlations
  - Table A.4. z Scores For a Few Selected Occupations
- Descriptive Statistics
  - Table A.5. Descriptive Statistics
- Additional Regression Results
  - Table A.6. Confidence: Binary Logistic Regression Results from Ballot B.
- References

**Measurement of the Occupation-level Variables**

***Complexity of Work***

I use data from the occupational requirements dimension of the O*NET content model to measure complexity of work. Nearly all O*NET concepts are measured by two survey questions. As an example, one concept is problem solving. Job incumbents are asked to rate on a five-point scale ‘how important it is to make decisions and solve problems to the performance of their current job.’ They are also asked on a seven-point scale what level of making decisions and solving problems is needed to perform their current job (examples to guide scoring on the level include: ‘determine the meal selection for a cafeteria,’ ‘select the location for a major department store,’ and ‘make the final decision about a company’s 5-year plan’). I began by multiplying these two scores (i.e., importance and level) together to make a single variable for each concept (i.e., a product variable). A high score on the product variable indicates both high importance and a high level.

I use these new product variables in exploratory factor analyses to measure several possible dimensions of work complexity: 1) complexity of information input, 2) complexity of mental processes, 3) complexity of interacting with others, and 4) independence (i.e., freedom from supervision). This list of possible dimensions of work complexity is drawn largely from the O*NET content model, but is also informed by the research of Kohn and his collaborators discussed in the article (e.g., Kohn and Slomczynski, 1993). Multiple items are available in the O*NET data to measure each of these possible dimensions of work complexity – ranging from two items for independence to seventeen items for interacting with others. Details for these items are listed in Table A.1. I used exploratory factor analysis and reliability analysis to construct a standardized variable for each possible dimension. The factor loadings and alpha levels from these analyses are listed in Table A.2.

Bivariate correlations between these standardized variables indicate the existence of multicollinearity (see Table A.3.). Collinearity diagnostics from preliminary regression models (not shown) indicated that this multicollinearity was degrading estimates. As a result, I further reduced these four possible dimensions into a single measure of work complexity – that is, I entered the four standardized variables into a factor analysis and saved the single, extracted factor as a new variable (Cronbach’s alpha=0.878). The factor loadings from this factor analysis are: information input (0.787), mental processes (0.963), interacting with others (0.914), and independence (0.741).

***Science Work***

One item is available in the ‘worker requirements – basic skills’ portion of the O*NET content model to measure science work. Similar to the other items described above, it includes separate measures for the importance and the level – that is, these indicate the level of ‘using scientific rules and methods to solve problems’ and the importance of ‘using scientific rules and methods to solve problems.’ Importance is measured on a five-point scale (ranging from not important to extremely important) while the level is measured on a seven-point scale. I constructed the science work variable by multiplying these two items together (making a product variable) and standardizing the result.

The bivariate correlation between work complexity and science work is 0.55. I list a few occupations and their z scores on the final science work and work complexity variables in Table A.4. There appears to be more variability in the degree of science work for occupations that are more complex – that is, some occupations scoring high on complexity do not require much science, while other occupations score quite high on both (e.g., compare emergency management directors and nurse anesthetists in Table A.4). This tends not to be true for occupations scoring lower on complexity. In general, the occupations scoring lower on complexity consistently require less science work.

***Table A.1. Item Details***

The following items (product variables) were used to construct the final measure of work complexity:

- Information input – 5 items
  - Looking for and receiving job-related information:
    - (Item 1) Observing, receiving, and otherwise obtaining information from all relevant sources.
    - (Item 2) Monitoring and reviewing information from materials, events, or the environment, to detect or assess problems.
  - Identifying and evaluating job-relevant information:
    - (Item 3) Identifying information by categorizing, estimating, recognizing differences or similarities, and detecting changes in circumstances or events.
    - (Item 4) Inspecting equipment, structures, or materials to identify the cause of errors or other problems or defects.
    - (Item5) Estimating sizes, distances, and quantities; or determining time, costs, resources, or materials needed to perform a work activity.
- Mental processes – 10 items
  - Information and data processing:
    - (Item 1) Assessing the value, importance, or quality of things or people.
    - (Item 2) Compiling, coding, categorizing, calculating, tabulating, auditing, or verifying information or data.
    - (Item 3) Using relevant information and individual judgment to determine whether events or processes comply with laws, regulations, or standards.
    - (Item 4) Identifying the underlying principles, reasons, or facts of information by breaking down information or data into separate parts.
  - Reasoning and decision making:
    - (Item 5) Analyzing information and evaluating results to choose the best solution and solve problems.
    - (Item 6) Developing, designing, or creating new applications, ideas, relationships, systems, or products, including artistic contributions.
    - (Item 7) Keeping up-to-date technically and applying new knowledge to your job.
    - (Item 8) Establishing long-range objectives and specifying the strategies and actions to achieve them.
    - (Item 9) Scheduling events, programs, and activities, as well as the work of others.
    - (Item 10) Developing specific goals and plans to prioritize, organize, and accomplish your work.

***Table A.1. Continued.***

- Interacting with others – 17 items
  - Communicating and interacting:
    - (Item 1) Translating or explaining what information means and how it can be used.
    - (Item 2) Providing information to supervisors, co-workers, and subordinates by telephone, in written form, e-mail, or in person.
    - (Item 3) Communicating with people outside the organization, representing the organization to customers, the public, government, and other external sources. This information can be exchanged in person, in writing, or by telephone or e-mail.
    - (Item 4) Developing constructive and cooperative working relationships with others, and maintaining them over time.
    - (Item 5) Providing personal assistance, medical attention, emotional support, or other personal care to others such as coworkers, customers, or patients.
    - (Item 6) Convincing others to buy merchandise/goods or to otherwise change their minds or actions.
    - (Item 7) Handling complaints, settling disputes, and resolving grievances and conflicts, or otherwise negotiating with others.
    - (Item 8) Performing for people or dealing directly with the public. This includes serving customers in restaurants and stores, and receiving clients or guests.
  - Coordinating, Developing, Managing, and Advising:
    - (Item 9) Getting members of a group to work together to accomplish tasks.
    - (Item 10) Encouraging and building mutual trust, respect, and cooperation among team members.
    - (Item 11) Identifying the educational needs of others, developing formal educational or training programs or classes, and teaching or instructing others.
    - (Item 12) Providing guidance and direction to subordinates, including setting performance standards and monitoring performance.
    - (Item 13) Identifying the developmental needs of others and coaching, mentoring, or otherwise helping others to improve their knowledge or skills.
    - (Item 14) Providing guidance and expert advice to management or other groups on technical, systems-, or process-related topics.
  - Administering:
    - (Item 15) Performing day-to-day administrative tasks such as maintaining information files and processing paperwork.
    - (Item 16) Recruiting, interviewing, selecting, hiring, and promoting employees in an organization.
    - (Item 17) Monitoring and controlling resources and overseeing the spending of money.

***Table A.1. Continued.***

- Independence (from supervision) – 2 items:
  - Criticality of position:
    - (Item 1) How much decision-making freedom, without supervision, does the job offer?
  - Routine versus Challenging Work:
    - (Item 2) To what extent is this job structured for the worker, rather than allowing the worker to determine tasks, priorities, and goals?

***Table A.2. Factor Loadings.^a^***

| Information input | | Mental processes | | Interacting with others | | Independence | |
| --- | --- | --- | --- | --- | --- | --- | --- |
| Item 1 | .592 | Item 1 | .705 | Item 1 | .672 | Item 1 | .902 |
| Item 2 | .897 | Item 2 | .617 | Item 2 | .782 | Item 2 | .902 |
| Item 3 | .794 | Item 3 | .802 | Item 3 | .742 |  |  |
| Item 4 | .368 | Item 4 | .846 | Item 4 | .789 |  |  |
| Item 5 | .699 | Item 5 | .899 | Item 5 | .326 |  |  |
|  |  | Item 6 | .709 | Item 6 | .546 |  |  |
|  |  | Item 7 | .868 | Item 7 | .783 |  |  |
|  |  | Item 8 | .849 | Item 8 | .369 |  |  |
|  |  | Item 9 | .774 | Item 9 | .838 |  |  |
|  |  | Item 10 | .832 | Item 10 | .886 |  |  |
|  |  |  |  | Item 11 | .678 |  |  |
|  |  |  |  | Item 12 | .867 |  |  |
|  |  |  |  | Item 13 | .856 |  |  |
|  |  |  |  | Item 14 | .841 |  |  |
|  |  |  |  | Item 15 | .721 |  |  |
|  |  |  |  | Item 16 | .836 |  |  |
|  |  |  |  | Item 17 | .703 |  |  |
|  |  |  |  |  |  |  |  |
| Cronbach’s Alpha | .775 |  | .941 |  | .940 |  | .897 |

a. Item details are presented in Table A.1. Factor loadings are from four separate exploratory factor analyses (one for each concept).

***Table A.3. Bivariate Correlations.^a^***

|  | (1) | (2) | (3) | (4) | (5) | (6) |
| --- | --- | --- | --- | --- | --- | --- |
| Information input (1) | 1.00 |  |  |  |  |  |
| Mental processes (2) | 0.74* | 1.00 |  |  |  |  |
| Interacting with others (3) | 0.61* | 0.88* | 1.00 |  |  |  |
| Independence (4) | 0.34* | 0.64* | 0.59* | 1.00 |  |  |
| Complexity of work (5) | 0.79* | 0.96* | 0.91* | 0.74* | 1.00 |  |
| Science work (6) | 0.62* | 0.56* | 0.38* | 0.33* | 0.55* | 1.00 |

* p < .05 (2-tailed)

a. These correlations were calculated after aggregating the occupation-level data to 2010 census codes and merging them with the person-level data from the General Social Survey. Information

input, mental processes, interacting with others, and independence were ultimately combined into the more general complexity of work variable.

***Table A.4. z Scores For a Few Selected Occupations.***

| Occupation (2010 Census Code) | Science Work (z) | Complexity of Work (z) |
| --- | --- | --- |
| Postal service mail carriers (5550) | -0.7 | -2.3 |
| Gaming services workers (4400) | -0.6 | -2.2 |
| Teacher assistants (2540) | -0.4 | -1.8 |
|  |  |  |
| Personal care aides (4610) | -0.3 | -1.1 |
| Model makers and pattern makers, metal and plastic (8060) | -0.2 | -1.1 |
| Bus drivers (9120) | -0.7 | -1.0 |
|  |  |  |
| Small engine mechanics (7240) | 0.1 | -0.7 |
| Drafters (1540) | 0.3 | -0.6 |
| Metal furnace operators, tenders, pourers, and casters (8040) | 0.1 | -0.5 |
|  |  |  |
| Flight attendants (9050) | -0.5 | 0.0 |
| Dental hygienists (3310) | 1.3 | 0.1 |
| Miscellaneous plant and system operators (8630) | 0.8 | 0.0 |
|  |  |  |
| Dispatchers (5520) | -0.5 | 0.5 |
| Nuclear engineers (1510) | 4.6 | 0.5 |
| Phlebotomists (3649) | 0.8 | 0.6 |
|  |  |  |
| Public relations and fundraising managers (60) | -0.4 | 1.0 |
| Construction managers (220) | 0.7 | 1.0 |
| Computer network architects (1106) | 0.2 | 1.1 |
|  |  |  |
| Nurse anesthetists (3256) | 3.4 | 2.1 |
| Emergency management directors (425) | 0.3 | 2.1 |
| First-line supervisors of firefighting and prevention workers (3720) | 0.9 | 2.3 |

**Descriptive Statistics**

***Table A.5. Descriptive Statistics.***

|  | Mean | Median | Mode | St. dev. | Min. | Max. | N |
| --- | --- | --- | --- | --- | --- | --- | --- |
| *Dependent variables* |  |  |  |  |  |  |  |
| Interest in science (z) | 0.00 | 0.00 | 1.40 | 1.00 | -2.38 | 1.40 | 1,170 |
| Science knowledge (%) | 67.20 | 72.73 | 81.82 | 21.29 | 0.00 | 100.00 | 1,154 |
|  |  |  |  |  |  |  |  |
| *Independent variables* |  |  |  |  |  |  |  |
| Work complexity (z) | 0.00 | -0.02 | -0.42 | 1.00 | -2.34 | 2.40 | 2,195 |
| Science work (z) | 0.00 | -0.38 | -0.67 | 1.00 | -0.67 | 5.66 | 2,195 |
| Years of schooling | 13.73 | 14 | 12 | 2.97 | 0 | 20 | 2,345 |
| Age (years) | 48.97 | 48 | 34 | 18.06 | 18 | 89 | 2,341 |
| Vocabulary test score | 5.91 | 6 | 6 | 2.02 | 0 | 10 | 1,547 |
|  |  |  |  |  |  |  |  |
|  |  |  |  |  |  |  |  |
|  | Frequency | Valid Percent |  |  |  |  |  |
| *Dependent variables* |  |  |  |  |  |  |  |
| Confidence in sci. comm. | 1,506 |  |  |  |  |  |  |
| Hardly any/only some | 825 | 54.8 |  |  |  |  |  |
| A great deal | 681 | 45.2 |  |  |  |  |  |
|  |  |  |  |  |  |  |  |
| *Independent variables* |  |  |  |  |  |  |  |
| Highest degree | 2,348 |  |  |  |  |  |  |
| Less than high school | 262 | 11.2 |  |  |  |  |  |
| High school | 1,178 | 50.2 |  |  |  |  |  |
| Associate’s degree | 196 | 8.3 |  |  |  |  |  |
| Bachelor’s degree | 465 | 19.8 |  |  |  |  |  |
| Graduate degree | 247 | 10.5 |  |  |  |  |  |
|  |  |  |  |  |  |  |  |
| Sex | 2,348 |  |  |  |  |  |  |
| Female | 1,296 | 55.2 |  |  |  |  |  |
| Male | 1,052 | 44.8 |  |  |  |  |  |
|  |  |  |  |  |  |  |  |
| Race and ethnicity | 2,348 |  |  |  |  |  |  |
| White | 1,512 | 64.4 |  |  |  |  |  |
| Black | 371 | 15.8 |  |  |  |  |  |
| Hispanic | 313 | 13.3 |  |  |  |  |  |
| Other | 152 | 6.5 |  |  |  |  |  |

***Table A.5. Continued.***

|  | Frequency | Valid Percent |  |  |  |  |  |
| --- | --- | --- | --- | --- | --- | --- | --- |
| Religious identity | 2,179 |  |  |  |  |  |  |
| Evangelical | 533 | 24.5 |  |  |  |  |  |
| Mainline | 277 | 12.7 |  |  |  |  |  |
| Black Protestant | 162 | 7.4 |  |  |  |  |  |
| Catholic | 493 | 22.6 |  |  |  |  |  |
| Jewish | 39 | 1.8 |  |  |  |  |  |
| Other faith | 133 | 6.1 |  |  |  |  |  |
| No faith | 542 | 24.9 |  |  |  |  |  |
|  |  |  |  |  |  |  |  |
| Political party | 2,315 |  |  |  |  |  |  |
| Democrat | 731 | 31.6 |  |  |  |  |  |
| Independent | 980 | 42.3 |  |  |  |  |  |
| Republican | 527 | 22.8 |  |  |  |  |  |
| Other party | 77 | 3.3 |  |  |  |  |  |
|  |  |  |  |  |  |  |  |
| Working full-time | 2,348 |  |  |  |  |  |  |
| No | 1214 | 51.7 |  |  |  |  |  |
| Yes | 1134 | 48.3 |  |  |  |  |  |

**Additional Regression Results**

***Table A.6. Confidence: Binary Logistic Regression Results from Ballot B (N=334).***

|  | Model 1 | | Model 2 | | Model 3 | |
| --- | --- | --- | --- | --- | --- | --- |
|  | Logit | Robust se | Logit | Robust se | Logit | Robust se |
| Highest degree (ref=less than HS) |  |  |  |  |  |  |
| High school | 0.660 | 0.580 | 0.574 | 0.578 |  |  |
| Junior college | 0.593 | 0.688 | 0.665 | 0.681 |  |  |
| Bachelor’s degree | 1.542* | 0.638 | 1.637* | 0.647 |  |  |
| Graduate degree | 1.297* | 0.694 | 1.660* | 0.722 |  |  |
| Years of education (centered) |  |  |  |  | 0.089 | 0.056 |
| Complexity of work (z score) |  |  | -0.078 | 0.171 | 0.017 | 0.164 |
| Science work (z score) |  |  | -0.127 | 0.173 | -0.083 | 0.169 |
| *Control variables* |  |  |  |  |  |  |
| Sex (male=1) | 0.536** | 0.261 | 0.589** | 0.266 | 0.528** | 0.259 |
| Age (Centered) | -0.017** | 0.008 | -0.014 | 0.008 | -0.011 | 0.008 |
| Race and ethnicity (ref=White) |  |  |  |  |  |  |
| Black | -1.381** | 0.506 | -1.367** | 0.514 | -1.233** | 0.499 |
| Hispanic | -0.027 | 0.429 | 0.007 | 0.448 | 0.032 | 0.421 |
| Other | -0.344 | 0.459 | -0.304 | 0.469 | -0.219 | 0.477 |
| Rel. identity (ref=Evangelical) |  |  |  |  |  |  |
| Mainline | 0.506 | 0.480 | 0.412 | 0.495 | 0.511 | 0.468 |
| Black Protestant | 1.396** | 0.605 | 1.407** | 0.607 | 1.267** | 0.602 |
| Catholic | 0.715 | 0.415 | 0.587 | 0.442 | 0.603 | 0.427 |
| Jewish | 0.813 | 0.893 | 0.715 | 0.833 | 0.878 | 0.883 |
| Other faith | 0.918 | 0.543 | 0.863 | 0.565 | 0.808 | 0.516 |
| No affiliation | 0.204 | 0.392 | 0.163 | 0.408 | 0.202 | 0.405 |
| Political party (ref=Democrat) |  |  |  |  |  |  |
| Independent | -0.238 | 0.290 | -0.215 | 0.299 | -0.213 | 0.299 |
| Republican | -0.798** | 0.391 | -0.706 | 0.397 | -0.651 | 0.375 |
| Other party | -1.327 | 0.793 | -1.218 | 0.835 | -1.344 | 0.855 |
| Working full-time (yes=1) | -0.056 | 0.279 | -0.056 | 0.298 | 0.007 | 0.284 |
| Vocabulary test score (centered) | 0.013 | 0.080 | 0.016 | 0.084 | 0.034 | 0.078 |
| Interest in science (z score) | 0.467** | 0.157 | 0.501** | 0.165 | 0.476** | 0.157 |
| Science knowledge (%) | 0.011 | 0.008 | 0.014 | 0.009 | 0.015 | 0.009 |
| Intercept | -2.145 | 0.909 | -2.320 | 0.933 | -1.606 | 0.732 |

***Table A.6. Continued.***

|  | Model 1 | | Model 2 | | Model 3 | |
| --- | --- | --- | --- | --- | --- | --- |
|  |  |  |  |  |  |  |
| N | 344 |  | 344 |  | 334 |  |
| Wald chi-square | 47.54 |  | 53.27 |  | 49.23 |  |
| DF | 22 |  | 24 |  | 21 |  |
| Prob > chi-square | 0.001 |  | 0.001 |  | 0.001 |  |
| Pseudo r-squared | 0.145 |  | 0.154 |  | 0.131 |  |
| Log pseudo-likelihood | -198.648 |  | -191.030 |  | -196.228 |  |

** p < 0.05 (one-tailed); ** p < 0.05 (two-tailed)*

**References**

Kohn ML and Slomczynski KM (1993) *Social Structure and Self-Direction: A Comparative Analysis of the United States and Poland*. Cambridge: Blackwell.

National Center for O*NET Development (2022) O*NET 24.2 Database. O*NET Resource Center. Available at: https://www.onetcenter.org/database.html (accessed 9 February 2022).
